# Dr. Aran

**Published:** 1861-05

**Authors:** 


DR. ARAN died late in February, from cerebral rheumatism, at the early age of forty-four. Dr. Aran was one of the most promising physicians of Paris, and a gentleman of undoubted merit and ability. He was deputy professor at the Faculty, and physician to the St. Antoine Hospital. He was the translator of Dr. Bennett's work on the diseases of the uterus, and had issued lectures on the same topic. Besides monographs on paralysis and diseases of the heart, he recently published his "Clinical Lectures on the Diseases of Women," which gained him an enviable reputation both at home and abroad.

